# Study of Disinfectant Resistance Genes in Ocular Isolates of *Pseudomonas aeruginosa*

**DOI:** 10.3390/antibiotics7040088

**Published:** 2018-10-15

**Authors:** Dinesh Subedi, Ajay Kumar Vijay, Mark Willcox

**Affiliations:** School of Optometry and Vision Science, University of New South Wales, Sydney, NSW 2052, Australia; d.subedi@unsw.edu.au (D.S.); m.willcox@unsw.edu.au (M.W.)

**Keywords:** *Pseudomonas aeruginosa*, disinfectant resistance, contact lens

## Abstract

Background: The prevalence of disinfectant resistance in *Pseudomonas aeruginosa* is on the rise. *P. aeruginosa* is the most common bacteria isolated from cases of microbial keratitis. Many multi-purpose contact lens disinfectant solutions are available to decontaminate contact lenses before use and to help reduce the incidence of infections. However, with increasing disinfectant resistance, the effect of multi-purpose disinfectant solutions may diminish. The goal of this study was to examine genes associated with disinfectant resistance in ocular isolates of *P. aeruginosa* and understand the strain’s susceptibility to different multipurpose disinfectant solutions. Methods: Seven potential disinfectant resistance genes were used in BLASTn searches against the whole genomes of 13 eye isolates of *P. aeruginosa*. A microdilution broth method was used to examine susceptibility to four different multipurpose disinfectant solutions. Results: All strains possessed the *sugE2*, *sugE3* and *emrE* (*qacE*) genes. The *sugE1* and *qacEdelta1* genes were present in 6/13 isolates. No strains contained the *qacF* or *qacG* genes. All tested disinfectant solutions had the ability to kill all test strains at 100% concentration, with some strains being susceptible at 1:8 dilutions of the disinfecting solutions. However, the presence of disinfectant resistance genes was not associated with susceptibility to multi-purpose disinfectants. Conclusion: All four tested contact lens disinfectant preparations are effective against *P. aeruginosa* isolates regardless of the presence of disinfectant resistance genes.

## 1. Introduction

*Pseudomonas aeruginosa* is a common isolate in eye infections [1]. A systematic review of *P. aeruginosa* in eye infections showed that about 20% of isolates in all eye infections are *P. aeruginosa* with the highest (68%) prevalence in contact lens associated keratitis (corneal ulcer) [2]. With wide array of pathogenic factors including exotoxins and proteases, *P. aeruginosa* keratitis is often fulminant and can rapidly destroy the cornea leading to vision loss [3]. This Gram-negative opportunistic bacterium utilises invasive and cytotoxic proteins to infect and damage corneal cells and cells of the immune system [4,5]. In addition, resistance to antibiotics and disinfectants has been commonly reported in clinical isolates of *P. aeruginosa* [6,7,8]. This leads to difficulties in treatment and ultimately poor outcomes of the disease [9]. Therefore, it is important to frequently monitor the status of antimicrobial resistance in *P. aeruginosa*.

Contaminated contact lenses are often associated with microbial keratitis [10]. Multi-purpose disinfectant solutions (MPDS) containing different combinations of disinfectants are used to clean and disinfect contact lenses and reduce contamination [11]. The most commonly used disinfectants in MPDS include quaternary ammonium compound (QAC) or biguanides, or both in different forms and concentrations [12]. Improper use of these solutions either intentionally or unintentionally, such as topping up or reuse of solution in the lens case, brings about exposure of bacteria to sub-lethal concentrations of disinfectants, and subsequently may help to develop resistance [13]. Consequently, many eye isolates have been reported to carry disinfectant resistance genes [14], which has been shown to be the cause for increased resistance to MPDS in *Staphylococcus aureus* [15]. However, the resistance rate to MPDS and associated genes in ocular *P. aeruginosa* are still not well studied.

At least seven genes (*sugE1*, *sugE2*, *sugE3*, *emrE* (*pae-qacE*), *qacEdelta1 qacF*, and *qacG*), which are the members of small multi drug resistance (SMR) protein, are associated with QAC resistance in *P. aeruginosa* [16,17]. Some of these genes are carried on integrons with other drug resistance genes. As a result of this, evidence of co-resistance to disinfectants in several bacterial species including *Staph. aureus* and *Acinetobacter* spp. have been found [14,18,19,20]. However, there have been no studies examining the susceptibility of contact lens disinfectants and associated resistance genes in ocular isolates of *P. aeruginosa*. We aimed to examine the profile of disinfectant associated resistance genes and correlate it with the susceptibility of four commonly available MPDS in Australia to *P. aeruginosa* isolates from eye infections. 

## 2. Results

### 2.1. Distribution of Disinfectant Resistance Genes

The *sugE2*, *sugE3* and *emrE* genes were found in all 13 isolates (Table 1). Whereas, *qacF* and *qacG* genes were not observed in any of the isolates. Two disinfectant resistance genes *sugE1* and *qacEdelta1* were present in 6 (46.2%) isolates (Table 1). Gene *sugE1* was observed in five isolates; PA31, PA32, PA33, PA35, PA37 and PA175 and *qacEdelta1* was observed in PA31, PA32, PA33, PA34, PA35 and PA37. It should be noted that these two genes were common in five isolates. (no *sugE1* in strain PA34, no *qacEdelta1* in strain PA175).

### 2.2. Susceptibility of P. aeruginosa to MPDS

The minimum inhibitory concentration (MIC) and minimum bactericidal concentration (MBC) of four commonly available MPDS for the thirteen ocular *P. aeruginosa* strains are shown in Table 2. OPTI-FREE^®^ Puremoist^®^ was most effective amongst four MPDS with median MIC and MBC 1:4, followed by renu^®^ fresh™ and RevitaLens Ocutech^®^ (median MIC = 1:4, median MBC = 1:2 each). Biotrue^®^ was the least effective and required four-fold higher concentration than OPTI-FREE^®^ Puremoist^®^ to kill 99.9% of *P. aeruginosa* strains. All tested MPDS had the ability to kill all test strains at 100% concentration. There were no associations between the presence of disinfectant resistance genes and MIC or MBC of MPDS.

## 3. Discussion

MPDS are commercial preparations containing a variety of ingredients including disinfectants and are used to prevent the growth of microbes on contact lenses [11]. Contaminated lenses are the main source of eye infections, including corneal ulcers that can develop rapidly and sometimes lead to vision loss [3]. *P. aeruginosa* is the major isolate from contact lens related infections and the antibiotic and disinfectant resistance in this species is on the rise [2]. We investigated the disinfectant resistance gene profile of ocular *P. aeruginosa* and susceptibility of these isolates to four MPDS to find the association between the presence of disinfectant resistance genes and susceptibility to MPDS. The MPDS inhibited the growth of all strains in the range from 1:2 dilution to in-use (100%) concentration. 46% of isolates possessed the *sugE1* and *qacEdelta1* disinfectant resistance genes.

Small multidrug resistance (SMR) proteins are drug-metabolite transporters and confer resistance to a variety of lipophilic compounds, such as quaternary ammonium compounds [21]. Increased expression of *sugE* in *E. coli* is associated with resistance to quaternary ammonium compounds [22]. Of the SMR encoding genes, the seven that have been frequently reported in *P. aeruginosa* [16], were used in BLASTn search to examine presence of disinfectant resistance genes in the studied strains. Two genes (*sugE1* and *qacEdelata1*) showed variations in their presence amongst 13 isolates. Interestingly, five *sugE1*+ isolates also carried the *qacEdelta1*. However, their presence was not significantly associated with the susceptibility to MPDS. *SugE1* has been identified in plasmids of many members of *Enterobacteriaceae* [23], and the other homologues (*sugE2* and *sugE3*) are associated with chromosomes [21]. The role of SUG proteins in disinfectant resistance *P. aeruginosa* has not been well explored. The *qacEdelata1* is associated with integrons which frequently carry antibiotic resistance genes [24]. Therefore, the presence of *qacEdelata1* is often correlated with antibiotic resistance. However, no associations were found between the presence of *qacE/qacEdelata1* and quaternary ammonium compounds resistance in Gram-negative bacteria including *P. aeruginosa* [25]. In contrast, *S. aureus* strains that possessed disinfectant resistance genes were more resistant to MPDS that those without such genes [15]. This highlights the possibility that disinfectant resistance genes are more active in Gram-positive bacteria, such as *S. aureus* than Gram negative bacteria, such as *P. aeruginosa*. However, our results are limited by the small sample size and this hypothesis requires further investigation with large numbers of strains.

The current study demonstrated that all four MPDS preparations were effective against the tested isolates. This may not be surprising given that all MPDS must pass the International Organisation for Standardisation test, ISO 14729, which includes testing for activity (≥3 log_10_ reduction in growth) against a standard strain of *P. aeruginosa* [26]. However, the current study did show that OPTI-FREE^®^ Puremoist^®^ was the most active against *P. aeruginosa*. The same MPDS has also been shown to have the highest activity against a common contact lens case isolate, *Stenotrophomonas maltophilia* [27]. However, when microbial contamination of contact lens cases was examined from people with keratitis cases; Biotrue^®^ had the least number of microbes [11]. The activity of MPDS depends on its composition and this includes disinfectants as well as surfactants and chelating agents (e.g. ethylenediaminetetraacetic acid; EDTA) [28]. Regular use of MPDS during contact lens care exposes the MPDS to organic compounds which can affect pH, osmolarity, and in situ concentrations. This could be the reason for observation of lower efficiency of OPTI-FREE^®^ Puremoist^®^ in the previous study [11].

All tested MPDS contained EDTA in different forms. EDTA is a metal chelator and actively disrupts the outer lipopolysaccharide layer of Gram-negative bacteria [29]. As a result, the membrane becomes more permeable to disinfectants. This may be a reason that resistance to disinfectant solutions was not detected in this study whereas resistance to quaternary ammonium compounds in *P. aeruginosa* has been frequently described elsewhere [30,31,32]. It should also be noted that the efficacy of disinfectants observed in in-vitro studies may not be obtained in in-situ conditions [33]. Further studies examining the resistance of *P. aeruginosa* strains to individual disinfectant ingredients of the MPDS may help to understand whether possession of certain genes leads to resistance. In addition, larger sample size that includes both contact and non-contact lens-related isolates might demonstrate significant association of disinfectant resistance genes with phenotypic resistance to disinfectants.

## 4. Materials and Methods

### 4.1. Bacterial Strains

Thirteen *P. aeruginosa* strains isolated from microbial keratitis were included in this study. These strains were isolated from various eye centres in Australia (*n* = 6) and India (*n* = 7). Cultures were preserved in tryptone soya broth (Oxoid, Basingstoke, UK) and 50% glycerol and stored at −80 °C. When required for assay, cultures were revived on tryptone soya agar (Oxoid) or nutrient agar (Oxoid) to obtain pure cultures.

### 4.2. Analysis of Disinfectant Resistance Genes

Seven genes (*sugE1*, *sugE2*, *sugE3*, *emrE* (*pae-qacE*), *qacEdelta1 qacF*, and *qacG*) that are associated with disinfectant resistance in *P. aeruginosa* were selected for screening [16,17]. The whole genome sequences of the strains were obtained, which had been made available in the NCBI database under Bio-project accession number PRJNA431326. To examine the presence of disinfectant resistance genes, a BLASTn search was performed against draft genome sequences of studied strains. Identity cut-off of 95%, that covered minimum 90% of the query, was taken as positive BLAST hit.

### 4.3. In-Vitro Susceptibility Testing of Multi-Purpose Disinfectant Solution (MPDS)

Four commercially-available MPDS for contact lenses were used to examine susceptibility (Table 3). A microdilution broth method, as described by the Clinical and Laboratory Standards Institute (CLSI, Wayne, PA, USA) [34], was used to determine MIC and MBC of each agent with some modifications as described elsewhere [27]. The bacterial suspension was prepared in Mueller-Hinton broth (MHB; Oxoid) and the final density of cells was adjusted to 1 × 10^8^ cfu/mL (OD_660_ 0.1). Each MPDS from commercial preparations was two-fold serially diluted in sterile deinonised water to obtain 1:2 (50%), 1:4 (25%), 1:8 (12.5%) 1:16 (6.25%) and 1:32 (3.12%) dilutions. Bacterial suspension of 20 µL was added into 200 µL of the serially diluted MPDS (in triplicate) into the wells of 96-well microtiter plates (Coaster Co., NY, USA) to maintain the final concentration of the inoculum at 1 × 10^7^ cfu/mL. The plates were incubated at 37 °C for 18–20 h and examined for turbidity in the spectrophotometer (660 nm). An aliquot of 10 µL from three wells of the concentrations, showing no visible growth of bacteria after incubation, was mixed with 90 µL of Difco^TM^ D/E Neutralising Broth (Becton Dickinson, Sparks, MD, USA) and used to plate on NA to obtain MBCs. MICs were taken as the lowest concentration of MPDS, which produced no turbidity (visible growth), and the MBC was taken as the lowest concentration of MPDS that inhibited 99.9% of the initial inoculum. All isolates were tested in triplicate and repeated twice.

## 5. Conclusions

All four tested contact lens disinfectant preparations are effective against *P. aeruginosa* isolates at 100% concentration, although they had varying efficiency at dilutions below this. *P. aeruginosa* strains had different disinfectant resistance gene profiles which indicates that each strain may have unique resistance mechanisms. This could affect the susceptibility to quaternary ammonium compounds, a common antimicrobial ingredient of MPDS. Therefore, it is essential to evaluate the efficacy of MPDS on a regular basis.

## Figures and Tables

**Table 1:** Distribution of different disinfectant resistance genes in *P. aeruginosa* (+ = gene presence; − = gene absence).

Strains	Disinfectant Resistance Genes						
	*sugE1*	*sugE2*	*sugE3*	*emrE* (*qacE*)	*qacEdelta1*	*qacF*	*qacG*
PA17	−	+	+	+	−	−	−
PA31	+	+	+	+	+	−	−
PA32	+	+	+	+	+	−	−
PA33	+	+	+	+	+	−	−
PA34	−	+	+	+	+	−	−
PA35	+	+	+	+	+	−	−
PA37	+	+	+	+	+	−	−
PA40	−	+	+	+	−	−	−
PA82	−	+	+	+	−	−	−
PA149	−	+	+	+	−	−	−
PA157	−	+	+	+	−	−	−
PA171	−	+	+	+	−	−	−
PA175	+	+	+	+	−	−	−

**Table 2:** Minimum inhibitory concentration and minimum bactericidal concentration of multipurpose disinfectant solutions.

Strains	Multipurpose Disinfectant Solution (Dilution Factor in Water)							
	OPTI-FREE^®^ Puremoist^®^		renu^®^ fresh™		Biotrue^®^		RevitaLens Ocutech^®^	
	MIC	MBC (≥99.9%)	MIC	MBC (≥99.9%)	MIC	MBC (≥99.9%)	MIC	MBC (≥99.9%)
PA17	1:4	1:4	1:4	1:2	1:2	1	1:4	1:2
PA31	1:8	1:4	1:2	1:2	1:2	1	1:4	1:2
PA32	1:4	1:4	1:4	1:2	1:2	1	1:4	1:2
PA33	1:8	1:4	1:2	1:2	1:2	1:2	1:4	1:2
PA34	1:8	1:4	1:4	1:2	1:4	1:2	1:4	1:2
PA35	1:4	1:2	1:4	1:2	1:2	1:2	1:4	1:2
PA37	1:8	1:4	1:4	1	1:2	1	1:4	1:2
PA40	1:4	1:4	1:4	1:2	1:2	1	1:2	1:2
PA82	1:8	1:4	1:4	1:2	1:4	1:2	1:8	1:2
PA149	1:4	1:4	1:4	1:2	1:4	1	1:4	1:2
PA157	1:4	1:4	1:2	1:2	1:4	1	1:4	1:2
PA171	1:4	1:4	1:4	1	1:2	1	1:4	1:2
PA175	1:8	1:4	1:4	1	1:4	1	1:8	1:2
**Median**	**1:4**	**1:4**	**1:4**	**1:2**	**1:2**	**1**	**1:4**	**1:2**

**Table 3:** Composition of multipurpose disinfectant solutions (MPDS) used in this study.

MPDS (Manufacturer)	Ingredients
Biotrue^®^ (Bausch & Lomb, Rochester, NY, USA)	Hyaluronan, sulfobetaine, poloxamine, boric acid, sodium borate, edetate sodium, sodium chloride, water, polyquaternium (POLYQUAD^®^; 0.0001%), polyaminopropyl biguanide (PHMB; 0.00013%)
OPTI-FREE^®^ Puremoist^®^ (Alcon Laboratories Inc., Forth Worth, TX, USA)	Sodium citrate, sodium chloride, boric acid, sorbitol, aminomethylpropanol, disodium EDTA, Tetronic^®^1304, EOBO-41^TM^ [polyethyleneoxide-polybutyleneoxide-41], water, Polyquaternium (POLYQUAD^®^; 0.001%), myristamidopropyl dimethylamine (ALDOX^®^; 0.0006%)
renu^®^ fresh™ (Bausch & Lomb, Rochester, NY, USA)	Hydroxyalkylphosphonate, boric acid, edetate disodium, poloxamine, sodium borate, sodium chloride, water DYMED^TM^ [Polyaminopropyl biguanide (PHMB; 0.0001%)]
RevitaLens Ocutech^®^ (Abbott Medical Optics Inc., Santa Ana, CA, USA)	Alexidine dihydrochloride (0.00016%), Polyquaternium-1 (0.0003%), boric acid, sodium borate decahydrate, edetate disodium, TETRONIC 904, sodium citrate, sodium chloride, water
